# The Evaluation of Smart Speaker Skills for Chronic Disease Management of Older Adults

**DOI:** 10.1093/geroni/igab046.2588

**Published:** 2021-12-17

**Authors:** Melissa Pighin, Yong Kyung Choi

**Affiliations:** 1 University of California, Davis, Sacramento, California, United States; 2 University of California Davis School of Medicine, Sacramento, California, United States

## Abstract

A voice-activated smart speaker is an emerging technology that presents unique opportunities to support the chronic disease management of older adults. We identified the available health-related smart speaker skills in Amazon Alexa platform that support chronic disease management and assessed their functionalities to inform the development of a home-based lifestyle intervention program for older adults with cardiovascular disease and type 2 diabetes. From January to March 2021, we searched Alexa Skills using keywords related to diabetes, medication, blood pressure and nutrition management. Our search produced total 156 potentially relevant skills (63 diabetes, 57 medication, 11 blood pressure and 25 nutrition related), of which 22 skills met inclusion criteria. Apps were excluded if it was only informational, not relevant to the topic, had zero user rating, available in language other than English, and required an external device or a subscription to a specific health plan or service. 22 skills (4 diabetes, 8 medication, 3 blood pressure and 7 nutrition) were evaluated with Echo Show 8 device. The skills were evaluated using the modified version of IMS Institute for Healthcare Informatics app functionality scores and the score (0 to 11) was calculated accordingly. The median number of functionalities was 3.5 and 68% of skills (15/22) had 4 or fewer functions. The highest rated skill was a medication management app named myNurseBot having 6 out of 11 functionalities. The poor functionality score highlights a need for a more robust and comprehensive smart speaker skill to support the health management of older adults.

